# Evaluation of variables associated with the patency of arteriovenous fistulas for hemodialysis created by a nephrologist

**DOI:** 10.1590/2175-8239-JBN-2017-0014

**Published:** 2018-08-02

**Authors:** Anderson Tavares Rodrigues, Fernando Antônio Basile Colugnati, Marcus Gomes Bastos

**Affiliations:** 1 Universidade Federal de Juiz de Fora, Juiz de Fora, MG, Brasil.; 2 Universidade Federal de Juiz de Fora, Faculdade de Medicina, Juiz de Fora, MG, Brasil.

**Keywords:** Arteriovenous Fistula, Renal Dialysis, Renal Insufficiency, Chronic, Vascular Patency, Fístula Arteriovenosa, Diálise Renal, Insuficiência Renal Crônica, Grau de Desobstrução Vascular

## Abstract

**Introduction:**

Arteriovenous fistula (AVF) is considered the gold standard vascular access for chronic hemodialysis, and its failure predicts higher morbidity and mortality rates.

**Objective:**

1) To evaluate the success rate of AVF created by a nephrologist and 2) to identify clinical, laboratory, and demographic variables that influence AVF patency.

**Methods:**

A retrospective cohort study of 101 patients with chronic kidney disease for a total of 159 AVF created by a nephrologist between June 2010 and June 2013. Results: Of the AVFs created, 124 (78%) displayed immediate patency and 110 (62.9%) displayed late patency. Hemoglobin (10-12 g/dL) was the only variable related to late AVF patency (*p* = 0.05). An elevated blood pressure at time of surgery was associated with a lower number of procedures per patient (*p* = 0.001). Proximal AVF occurred more frequently in patients with dual access (*p* = 0.03). The AVF success rate was similar to those previously reported in the literature.

**Conclusion:**

Hemoglobin level in the recommended range has a favorable impact on late AVF patency and elevated blood pressure during surgery on the lower number of vascular accesses per patient. The high success rate indicates that it can be placed by trained nephrologists.

## RESUMO

**Introdução:**

A fístula arteriovenosa (FAV) é considerada o acesso vascular padrão ouro para hemodiálise crônica, e sua falha prediz taxas mais altas de morbimortalidade.

**Objetivos:**

1) Avaliar a taxa de sucesso da FAV criada por um nefrologista e 2) identificar variáveis clínicas, laboratoriais e demográficas que influenciam a patência da FAV.

**Métodos:**

Estudo de coorte retrospectivo de 101 pacientes com doença renal crônica, totalizando 159 FAVs criados por um nefrologista entre junho de 2010 e junho de 2013. Resultados: Das FAVs criadas, 124 (78%) apresentaram patência imediata e 110 (62,9%) apresentaram patência tardia. A hemoglobina (10-12 g/dL) foi a única variável relacionada à patência tardia da FAV (*p* = 0,05). A pressão arterial elevada no momento da cirurgia foi associada a um menor número de procedimentos por paciente (*p* = 0,001). A FAV proximal ocorreu com maior frequência em pacientes com dois acessos confeccionados (*p* = 0,03). A taxa de sucesso da FAV foi semelhante à relatada anteriormente na literatura.

**Conclusão:**

O nível de hemoglobina no intervalo recomendado tem um impacto favorável na perviedade tardia da FAV e pressão arterial elevada durante a cirurgia no menor número de acessos vasculares por paciente. A alta taxa de sucesso indica que ela pode ser feita por nefrologistas treinados.

## INTRODUCTION

Placement of the autogenous arteriovenous fistula (AVF) on the wrist as initially described in 1966^1^ remains the vascular access of choice for the hemodialysis treatment since it is associated with low levels of complications and better long-term durability.^2^ In addition, the autogenous AVF is associated with a lower mortality rate in the first 6 months of hemodialysis compared to the use of central venous catheters and vascular access with prosthesis.^3^ In 2016 in Brazil, the prevalence of dialysis patients using central venous catheter as vascular access was 20.5%, and 2.2% were using prosthesis, meaning that 77.3% had autogenous AVF.^4^

AVF failure is an important clinical problem with high cost and morbidity in the treatment of patients with chronic kidney disease (CKD) stage 5 on hemodialysis.^5^ The expenses with vascular access for hemodialysis are high; for example, in 2007 in the United States, the Medicare expenditures for vascular access for dialysis were around US$1.8 billion.^6^ Vascular access failure for hemodialysis significantly increases morbidity rates and treatment costs,^7^ while functioning AVF are associated with lower mortality rates.^8^

In the United States, vascular access is created mainly by the vascular surgeon in contrast with that of other countries in which the nephrologist plans and creates the AVF.^9^ For example, in Italy and Japan, the rates of AVF placement by the nephrologist are 85% and 25%, respectively. In these countries, the time between the indication for and the creation of vascular access is 5-6 days, much lower than the 40-43 days observed in Canada and the United Kingdom, countries in which AVF are created by surgeons. As a result, this delay influenced the proportion of patients who started dialysis with an AVF.^7^

To date, no publication has reported on the experience of creating AVF by Brazilians nephrologists, thereby justifying the studies that assess this possibility as a strategy to reduce the use of central venous catheters at the start of dialysis. Thus, the objectives of this study were to evaluate the immediate and late patency rates of AVF created by a nephrologist and identify the demographic, clinical, and laboratory variables associated with vascular access functionality.

## METHODOLOGY

### SAMPLE AND SETTINGS

This is a retrospective cohort study involving patients from the *Pró Renal Centro de Nefrologia* clinic, Barbacena, Minas Gerais, Brazil, on chronic hemodialysis who provided consent for the construction of an AVF by a nephrologist (A.T.R.) who was previously trained to perform the procedure. Since there is no formal curriculum in nephrology for the training of the creation of AVF, a fellowship training was took by the author (A.T.R.) with a vascular surgeon during a period of three months, when around 100 fistulas were made.

The evaluated period included the start of AVF creation by the nephrologist in June 2010 until June 2013. Patients whose AVF were created by other medical professionals not belonging to the service were not included in the study.

The variables assessed were present at the time of AVF placement since the same patient may have undergone more than one procedure. The information was transcribed into a database that was specifically developed for the study and age, sex, and diagnosis of diabetes *mellitus* (DM) were recorded. We also analyzed the following variables: frequencies of the procedures per period of grouped months, the anatomic area where the procedure was performed, total number of procedures per patient, presence or absence of high blood pressure (BP) (defined as BP > 140/90 mmHg) at the time of surgery, levels of intact parathyroid hormone (iPTH), phosphorus (P), and hemoglobin (Hb), and the product of calcium (Ca) × P. All of the laboratory data were collected in the month of the access placement. The iPTH values were stratified into three groups: < 500 pg/dL, 501-1000 pg/dL, and > 1000 pg/dL; the product of Ca × P was divided into ≤ 55 and > 55; the level of P was divided into ≤ 5.5 mEq/L and > 5.5 mEq/L; the Hb values were stratified into ≤ 10 g/dL, 10.1-12.0 g/dL, and > 12 g/dL. The rate of complications related to the total number of procedures performed was also described.

The anatomical areas of the distal procedures were defined as those below the elbow fold with only one vascular anastomosis and using the radial or ulnar arteries and cephalic or basilic veins of the forearm, while those of the proximal procedures were defined as those in the region of the elbow fold with only one vascular anastomosis and using the brachial artery and basilic, cephalic, or brachial plexus veins.

Immediate patency was identified if the AVF presented thrill and/or palpable pulse and/or an audible murmur in the drainage vein in the immediate postoperative period and late patency if the AVF could be punctured and used for a hemodialysis session 30-60 days after the surgical procedure.

The study protocol was approved by the Committee for Ethics in Research of the Universidade Presidente Antônio Carlos - UNIPAC under protocol number 831/2010.

## STATISTICAL ANALYSIS

The data analysis was performed using the software IBM SPSS Statistics for Macintosh, Version 21.0 (Armonk, NY: IBM). The study variables corresponded to those registered in the database mentioned above. The frequency and the averages, standard deviations, and percentages indicated for each variable were calculated. The patency rates of the fistulae with the other study variables were made in row by column contingency tables or analysis of variance (ANOVA) tables. The statistical significance of the differences obtained in the comparisons was determined using the chi-squared test or Fisher's exact test for ANOVA tables. The dependent variables - immediate and late patency - were evaluated using logistic regression models and adjusted for high BP at the time of the procedure, Hb level, and number of procedures per patient to obtain the odds ratio (OR), 95% confidence interval (CI 95%), and P value. The level of statistical significance for the analysis was 5%.

## RESULTS

We studied 101 patients (49 men [48.5%], 52 women [51.5%]; 38 [37.6%] with DM as a CKD cause or a comorbidity) with a mean age of 56.3 ± 16.2 years. Of the 159 AVFs, 54 (34%) were created between June 2010 and June 2011, 53 (33.3%) between July 2011 and June 2012, and 52 (32.7%) between July 2012 and June 2013. The AVFs were created once in 100 patients (62.8%), twice in 38 (23.9%), and three or more times in 21 (13.2%). The maximum AVF per patient was five. An elevated BP was observed in 92 (58.6%) surgeries. The distal site was used in 95 (59,7%) procedures. Immediate and late AVF patency occurred in 124 (78.0%) and 110 (69.2%) procedures, respectively.

Table 1 presents the clinical and laboratory variables during AVF placement with immediate patency. As can be observed, it was not influenced by gender, diabetic status, different periods of AVF placement, number of procedures per patient, anatomical area of the fistula, or iPTH and P level. On the other hand, it is evident that the high BP, the product of Ca × P ≤ 55, and Hb levels of 10.1-12.0 g/dL tend to favor immediate AVF patency.

**Table 1:** Characteristics inherent to the fistula procedures that presented or not immediate patency

Characteristic	With immediate patency		Without immediate patency		χ^2^/F	*p*
	N	%	N	%		
Sex						
Male	59	47.6	19	54.3	0.491	0.567
Female	65	52.4	16	45.7		
Diabetes *mellitus*						
Yes	48	38.7	15	42.9	0.196	0.698
Not	76	61.3	20	57.1		
Procedural period						
June 2010 to June 2011	42	33.8	12	34.3	1.270	0.535
July 2011 to June 2012	39	31.5	14	40.0		
July 2012 to June 2013	43	34.7	9	25.7		
Nº. of procedures/patient						
1	80	64.5	20	57.1	3.478^a^	0.182
2	31	25.0	7	20.0		
≥ 3	13	10.5	8	22.9		
Elevated BP in surgery						
Yes	76	62.3	16	45.7	3.082	0.084
No	46	37.7	19	54.3		
Place of access						
Distal	75	62.0	20	58.8	0.112	0.842
Proximal	46	38.0	14	41.2		
iPTH level						
< 500	73	68.2	21	72.4	0.239^a^	0.948
501-1000	19	17.8	4	13.8		
> 1000	15	14.0	4	13.8		
Product of Ca × *p*						
≤ 55	70	66.7	24	85.7	3.870	0.062
> 55	35	33.3	4	14.3		
*p*						
≤ 5.5	51	48.6	18	64.3	2.187	0.201
> 5.5	54	51.4	10	35.7		
Hemoglobin						
≤ 10.0	70	69.3	20	76.9	4.596^a^	0.087
10.1-12.0	24	23.8	2	7.7		
> 12.0	7	6.9	4	15.4		

χ^2^, Pearson's chi-square; F, Fisher's exact test (a); BP, blood pressure; iPTH, intact parathyroid hormone level; Ca, calcium level; P, phosphorus level.

Table 2 shows the clinical and laboratory variables in relation to late AVF patency. An Hb level of 10.1-12.0 g/dL was the only variable that positively impacted late AVF patency.

**Table 2:** Characteristics inherent to the fistula procedure that presented or not late patency

Characteristic	With late patency		Without late patency		χ^2^/F	*p*
	N	%	N	%		
Sex						
Male	52	47.3	26	53.1	0.455	0.607
Female	58	52.7	23	46.9		
Diabetes *mellitus*						
Yes	44	40.0	19	38.8	0.021	1.000
No	66	60.0	30	61.2		
Procedural period						
June 2010 to June 2011	38	34.5	16	32.6	1.035	0.589
July 2011 to June 2012	34	30.9	19	38.8		
July 2012 to June 2013	38	34.5	14	28.6		
Nº. of procedures/patient						
1	69	62.7	31	63.3	0.884	0.631
2	28	25.5	10	20.4		
≥3	13	11.8	8	16.3		
Elevated BP in surgery						
Yes	67	62.0	25	51.0	1.686	0.131
No	41	38.0	24	49.0		
Place of access						
Distal	63	58.3	32	68.1	1.313	0.167
Proximal	45	41.7	15	31.9		
iPTH (pcg/dL)						
< 500	65	69.1	29	69.0	0.007	1.000
501-1000	16	17.1	7	16.7		
> 1000	13	13.8	6	14.3		
Product of Ca × P						
≤ 55	65	69.1	29	74.4	0.361	0.677
> 55	29	30.9	10	25.6		
P (mEq/L)						
≤ 5.5	48	51.1	21	53.8	0.085	0.850
> 5.5	46	48.9	18	46.2		
Hemoglobin (g/dL)						
≤ 10.0	61	67.7	29	78.4	5.998	0.050
10.1-12.0	23	25.6	3	8.1		
> 12.0	6	6.7	5	13.5		

χ^2^, Pearson's chi-square; F, Fisher's exact test (*); BP, blood pressure; iPTH, intact parathyroid hormone level; Ca, calcium level; P, phosphorus level.

Tables 3 and 4 present the OR of the logistic regression model adjusted for high BP at the time of surgery, number of procedures per patient (reference value equal to one procedure), and Hb (reference value, 10.1-12.0 g/dL); the dependent variables were immediate and late patency. There was a clear trend for high BP at time of AVF placement with immediate patency of the AVF (Table 3) and the positive impact of Hb levels of 10.1-12.0 g/dL in late patency.

**Table 3:** Results of the regression model applied to the dependent variable of immediate patency

Variable	Odds ratio	*p* value	95% CI
Elevated BP in surgery	2.66	0.055	0.98-7.21
No. of procedures/patient			
2	1.33	0.622	0.43-4.10
≥ 3	0.52	0.293	0.15-1.76
Hemoglobin (g/dL)			
≤ 10.0	0.23	0.071	0.05-1.13
> 12.0	0.19	0.094	0.03-1.33

CI, confidence interval; BP, blood pressure.

**Table 4:** Results of the regression model applied to the dependent variable of maintained late patency

Variable	Odds ratio	*p* value	95% CI
Elevated BP in surgery	1.45	0.396	0.61-3.45
No. of procedures/patient			
2	1.63	0.332	0.61-4.34
≥ 3	0.85	0.790	0.27-2.72
Hemoglobin (g/dL)			
≤ 10.0	0.25	0.035	0.07-0.90
> 12.0	0.17	0.043	0.03-0.94

CI, confidence interval; BP, blood pressure.

Regarding the procedural complications, two patients (1.25%) had ruptured pseudoaneurysm, three AVF were associated with vascular access steal syndrome (1.88%), and one patient (0.62%) presented infection with good response to treatment with short-spectrum antibiotics.

## DISCUSSION

Although our study was retrospective and carried out in a single center, which limits the generalizability of its results, its importance stems from being the first report of the experience of AVF created by a nephrologist in Brazil. The Brazilian renal replacement therapy data indicate an increasing number of patients with functional renal failure in need of dialysis (more than doubled in the last decade) and that a significant proportion of these patients start their treatment through temporary vascular access.^4^ Thus, our results in the Brazilian dialysis scenario are encouraging because they point to the possibility of nephrologists being empowered to construct AVF.

There is published evidence that the experience of the physician who creates AVF has a positive impact on its patency. For example, Fassiadis et al. observed an association between the experience of the surgeon that created the radiocephalic fistulae and the success of the procedure.^10^ In our study, after dividing the construction of fistulas in three consecutive periods, we found that the AVF success rate was equal in the three periods and was not associated with the nephrologist's surgical experience.

Blood pressure is an interesting aspect during AVF creation. In the dialysis clinic, it is not uncommon to observe AVF failure due to hypotensive episodes. A secondary analysis of the HEMO study^6^ demonstrated, for the first time, an association among pre-, intra- and post-dialytic hypotension and AVF loss. In our patients, high BP (> 140/90 mmHg) presented a protective effect in immediate AVF patency with a decreased need for new procedures per patient, but this did not correlate with late patency. As described by Gandhi et al., elevated BP was not associated with complications and presented as a safe issue for the patient undergoing vascular access procedure.^11^ We did not specifically analyze the BP level but tabulated only whether the patient was hypertensive or not at the time of surgery as qualitative data and not for quantitative analysis. However, our opinion is that asymptomatic hypertensive patients should not be treated. In further studies, we aim to analyze which level of PA is more favorable in promoting better access patency.

The non-uniformity of the distribution of sex in published studies limits the assessment of possible associations between sex and AVF patency. Although Wang et al. (2008)^12^ observed that their female patients had higher patency failure of the brachiocephalic fistulae, the AVF success rate was equal for the sexes, a result similar to ours.

The association between age and AVF patency remains controversial, with studies in favor^9^ and against,^12^ although elderly patients undergo hemodialysis treatment through AVF less often.^13^ Among our patients, only 27% were elderly, which does not allow us to infer the impact of age on AVF patency.

Diabetes *mellitus* is admittedly a risk factor for endothelial disease and, thus, potentially favors the failure rate of AVF. However, in agreement with the findings of Kazemzadeh et al. (2008),^14^ we did not observe a negative impact of DM on AVF patency in our patients.

The guidelines on vascular access of the Kidney Disease Outcomes Quality Initiative (KDOQI) in patients with CKD on dialysis treatment^15^ recommends distal AVF placement in the upper extremities to increase the possibility of creating new fistulae if needed. In our study, most AVF were placed distally (58.6%), while the ones placed proximally were associated with the need for more than one procedure.

The levels of iPTH and P as well as the product of Ca × P are associated with vascular changes such as calcification,^16^ atherosclerosis,^17^ and myointimal hyperplasia^18^ singly or in combination, and may negatively impact AVF patency. In our patients, at the construction of the AVF, the PTH, P, and Ca × P values were found within clinical limits recommended in the majority of the cases and did not influence the AVF patency outcome.

Recent studies performed in patients with non-dialysis-dependent CKD, showed a generally negative impact of the correction of Hb levels to normality in the cardiovascular mortality.^19^^,^ 20 On the other hand, fewer studies have correlated AVF patency and Hb level, but one reported that the patients with Hb levels of 10.1-12.0 g/dL had the highest fistula patency rates.^21^ In our study, although records of Hb values were absent in approximately 20% of patients at the time of vascular access placement, the OR of late AVF patency was also higher if the Hb level was 10.1-12 g/dL, while values < 10.0 g/dL and > 12 g/dL adversely affected venous access success.

As previously mentioned, in Brazil, the incidence and prevalence of patients who use the temporary central venous access for hemodialysis is unacceptably high.^4^ There are several reasons to explain this unfortunate observation: 1) late referral of patients with advanced CKD to the nephrologist; 2) inadequate AVF funding; and 3) in most renal replacement therapy programs, AVF are placed by vascular surgeons who have little time away from their practices to create AVF in view of the growing number of new patients with the indication for hemodialysis. Thus, it is important to evaluate whether nephrologists can be empowered to construct AVF for their patients. The experiences reported in other countries suggest that this is possible. For example, Garcia-Trío et al*.* found no difference in patency rates of AVF placed by nephrologists and surgeons and reported a 21.8% primary failure rate.^22^ In our study, 78.0% of AVF presented criteria of immediate patency, very similar to these data. In addition, 69.2% of our patients had AFV with late patency, i.e., fistulae suitable for venous puncture, a percentage similar to that observed in other studies.^23^^,^ 24 These findings are of great importance because they indicate a new possibility in the approach to AVF, since the nephrologists can prepare and create the AVF for their patients and, therefore, shortening the time to venous access placement compared to the long time still seen nowadays when the procedure is performed by surgeons.^7^ Also, it may become an important strategy to formalize a training curriculum within the scope of interventional nephrology in order to increase this excellent field of activity still not available to the Brazilian nephrologists.

Finally, it is important to emphasize the low rates of complications related to AVF in our patients. The percentage of complications observed is in accordance with published data.^25^ No severe early or late complication, which could cause consequences or permanent damage to patients, was observed. This reflects the importance of a good surgical technique and the professional's experience in the safe placement of AVF, avoiding complications, and obtaining good final results.

In conclusion, this retrospective study on AVF placement by a Brazilian nephrologist indicated a late patency success rate similar to nephrologists and surgeons in other countries. In addition, high BP (> 140/90 mmHg) is important for the immediate patency of venous access, and Hb levels of 10.1-12.0 g/dL is recommended to ensure late AVF patency.
